# Supplementary material for: A Retrospective Evaluation of the Predictive Value of Newborn Screening for Vitamin B12 Deficiency in Symptomatic Infants Below 1 Year of Age
Source: Int J Neonatal Screen. 2022 Dec 14;8(4):66. doi: 10.3390/ijns8040066 (PMC9782899; doi:10.3390/ijns8040066)
Supplement: Supplementary file 1 [file IJNS-08-00066-s001.zip › Caption Figure S1_ver2.pdf]

Figure S1. Results from applying the Austrian and Heidelberg algorithms for B12 deficiency on 70 clinical cases (black) and on a subgroup (orange) with s-vitamin B12<160 pmol/L or s-holotranscobalamin<35 pmol/L. Cutoffs in percentiles except for C3>10 µmol/L, ratios in %. Met=methionine, Phe=phenylalanine, C0=carnitine, C2=acetylcarnitine, C3=propionylcarnitine, C16=palmitoylcarnitine, tHcy=total homocysteine, MMA=methylmalonic acid, B12D=vitamin B12 deficiency. Adapted from (5,6).
