# Supplementary material for: A Retrospective Evaluation of the Predictive Value of Newborn Screening for Vitamin B12 Deficiency in Symptomatic Infants Below 1 Year of Age
Source: Int J Neonatal Screen. 2022 Dec 14;8(4):66. doi: 10.3390/ijns8040066 (PMC9782899; doi:10.3390/ijns8040066)
Supplement: Supplementary file 1 [file IJNS-08-00066-s001.zip › Table S1_ver2.pdf]

Table S1. Proportions of single metabolites and ratios above cutoff limits, and results from applying algorithms according to the Austrian and Heidelberg NBS for cases and controls (n, %).

	Clinical cases (n=70)	Clinical cases with B12<160 or holoTC<35 pmol/L (n=30)	Matched controls (n=646)	Un-matched controls (n=434)
C3>97p (4.0 µmol/L)	1 (1.4%)	1 (3.3%)	9 (1.4%)	28 (6.5%)
C3>99p (4.9 µmol/L)	1 (1.4%)	1 (3.3%)	4 (0.6%)	10 (2.3%)
MET<5p (11.7 µmol/L)	5 (7.1%)	3 (10%)	39 (6.0%)	43 (10%)
MET<0.59p (8.9 µmol/L)	1 (1.4%)	1 (3.3%)	6 (0.9%)	8 (1.8%)
MET/PHE<1.75p (0.21)	3 (4.3%)	2 (6.7%)	21 (3.3%)	21 (4.8%)
MET/PHE<5p (0.23)	5 (7.1%)	4 (13%)	39 (6.0%)	34 (7.8%)
MET/PHE>89p (0.44)	5 (7.1%)	3 (10%)	31 (4.8%)	13 (3.0%)
C3/C0>99.3p (0.29)	0	0	5 (0.8%)	11 (2.5%)
C3/C2>99.5p (0.21)	1 (1.4%)	1 (3.3%)	1 (0.2%)	4 (0.9%)
C3/C2>99.9p (0.25)	0	0	1 (0.2%)	0
C3/C16>99.2p (1.63)	2 (2.9%)	1 (3.3%)	6 (0.9%)	1 (0.2%)
C3/MET>97p (0.24)	3 (4.3%)	2 (6.7%)	11 (1.7%)	41 (9.4%)
tHcy>6.3 µmol/L	33 (47%)	17 (57%)	195 (30%)	46 (11%)
tHcy>8.6 µmol/L	13 (19%)	7 (23%)	60 (9.3%)	14 (3.2%)
MMA>0.423 µmol/L	10 (14%)	4 (13%)	22 (3.4%)	115 (26%)
MMA>2.35 µmol/L	1 (1.4%)	0	1 (0.2%)	0
MCA>0.34 µmol/L	2 (2.9%)	1 (3.3%)	31 (4.8%	7 (1.6%)
Heidelberg Met pathway	12 (17%)	8 (27%)	89 (14%)	72 (17%)
Heidelberg Met pathway and tHcy>8.6	3 (4.3%)	3 (10%)	4 (0.6%)	1 (0.2%)

Heidelberg C3 pathway	1 (1.4%)	1 (3.3%)	4 (0.6%)	14 (3.2%)
Heidelberg C3 pathway and MMA/MCA	0	0	0	2 (0.5%)
Austrian low C3 pathway	1 (1.4%)	1 (3.3%)	6 (0.9%)	15 (3.5%)
Austrian low C3 and tHcy>6.3	1 (1.4%)	1 (3.3%)	3 (0.5%)	1 (0.2%)
Austrian low C3 and tHcy>8.6	0	0	1 (0.2%)	1 (0.2%)
Austrian high C3 pathway	1 (1.4%)*	1 (3.3%)*	4 (0.6%)*	10 (2.3%)*
Austrian high C3 and tHcy>6.3	1 (1.4%)*	1 (3.3%)*	2 (0.3%)*	1 (0.2%)*
Austrian high C3 and tHcy>8.6	0	0	1 (0.2%)*	1 (0.2%)*
Austrian Met pathway	3 (4.3%)	2 (6.7%)	23 (3.6%)	24 (5.5%)
Austrian Met and tHcy>6.3	1 (1.3%)	1 (3.3%)	4 (0.6%)	0
Austrian Met and tHcy>8.6	0	0	0	0

*=overlapping with low C3 pathway, Met=methionine, Phe=phenylalanine, C0=carnitine, C2=acetylcarnitine, C3=propionylcarnitine, C16=palmitoylcarnitine, tHcy=total homocysteine, MMA=methylmalonic acid, MCA=methylcitric acid.
