# Supplementary figures and images for: A Retrospective Evaluation of the Predictive Value of Newborn Screening for Vitamin B12 Deficiency in Symptomatic Infants Below 1 Year of Age
Source: Int J Neonatal Screen. 2022 Dec 14;8(4):66. doi: 10.3390/ijns8040066 (PMC9782899; doi:10.3390/ijns8040066)

## Austrian algorithm

## Heidelberg algorithm

1<sup>st</sup> tiers

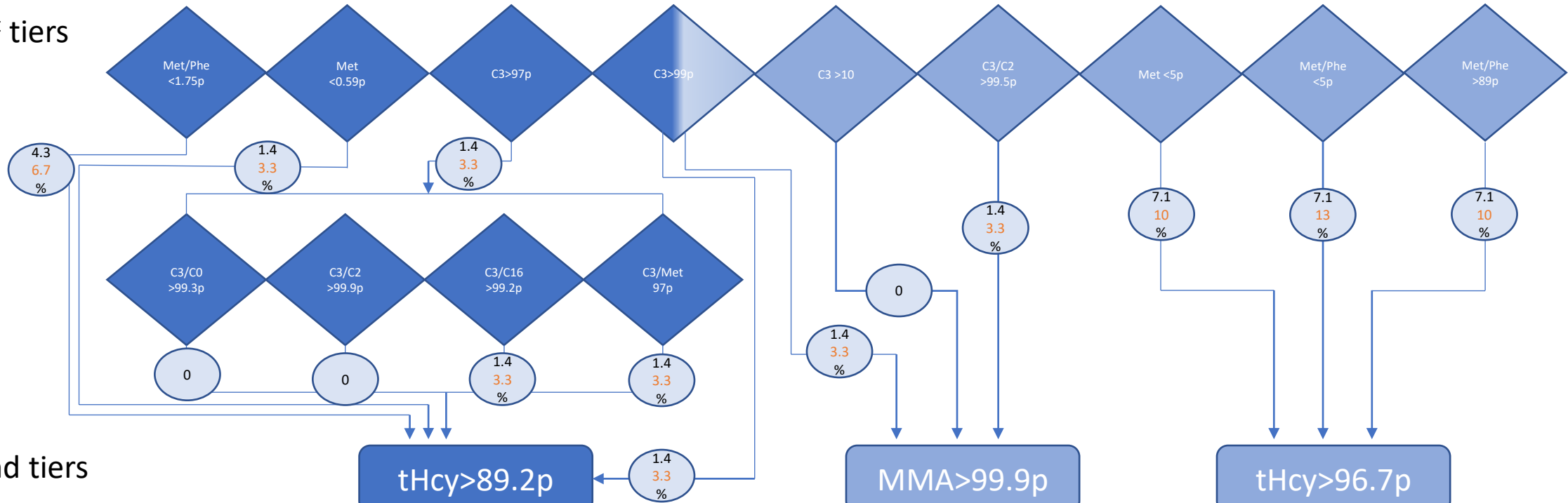

2nd tiers

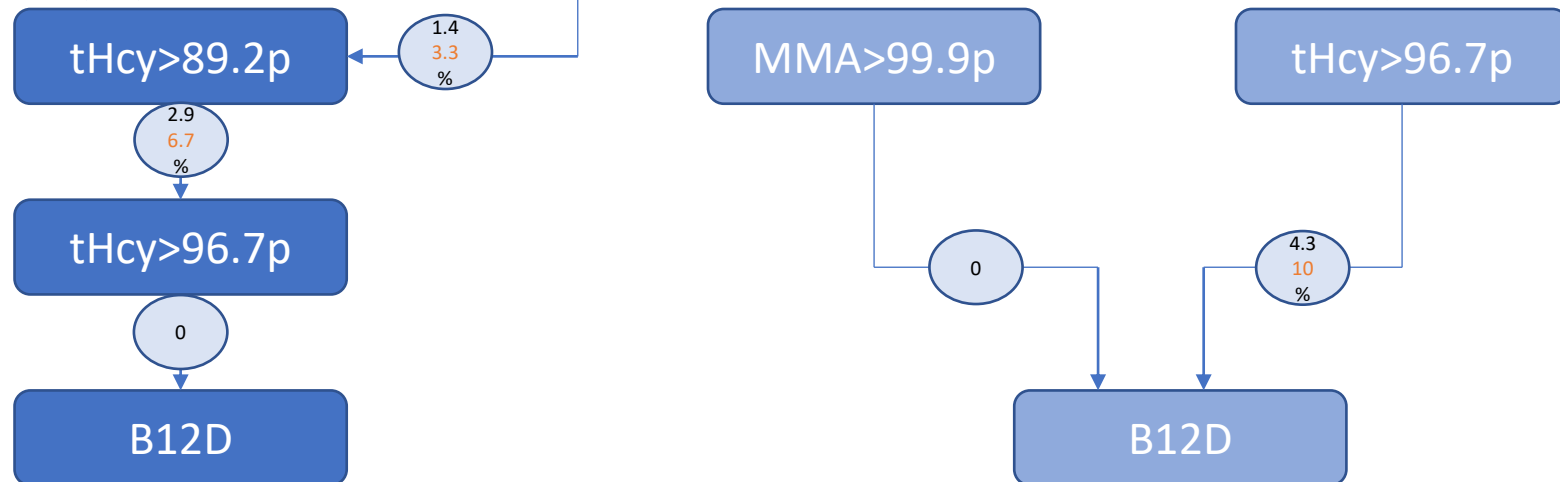

Supplement: Supplementary file 1 [file IJNS-08-00066-s001.zip › Figure S1_ver2.pdf]
